# LONGITUDINAL CHANGES IN DAILY STRESS REACTIVITY AND FUNCTIONAL HEALTH ACROSS 20 YEARS OF ADULTHOOD

**DOI:** 10.1093/geroni/igad104.1012

**Published:** 2023-12-21

**Authors:** Jonathan Rush, Emily Willroth, Eric Cerino, Jennifer Piazza, Susan Charles, Daniel Mroczek, David Almeida

**Affiliations:** University of Victoria, Victoria, British Columbia, Canada; Washington University in St. Louis, St. Louis, Missouri, United States; Northern Arizona University, Flagstaff, Arizona, United States; California State University, Fullerton, Fullerton, California, United States; University of California, Irvine, Irvine, California, United States; Northwestern University, Chicago, Illinois, United States; The Pennsylvania State University, State College, Pennsylvania, United States

## Abstract

The daily within-person association between stress exposure and negative affect (i.e., stress reactivity) has been shown to be predictive of a number of adverse health outcomes (e.g., inflammation, chronic conditions). These findings typically rely on a single burst of daily assessments. Little is known about the influence of long-term changes in daily stress reactivity across adulthood and its association with changes in health outcomes. The present study examined whether longitudinal changes in daily within-person associations between stress and affect over 20 years predicted long-term changes in functional health. We used measurement burst data from the National Study of Daily Experiences subsample (N=2,880) embedded within the MIDUS longitudinal study. Three measurement bursts were separated by ten years, with each containing daily measures of stress and affect across eight consecutive days, yielding 33,942 days of data across 20 years of adulthood. Functional health was measured by basic and instrumental activities of daily living (BADL; IADL) at three measurement waves spanning 20 years. Multilevel structural equation models were fit to simultaneously model short-term within-person associations between stress and affect (i.e., stress reactivity) at Level 1; long-term changes in stress reactivity at Level 2; and the association between changes in stress reactivity and changes in functional health at Level 3. Changes in stress reactivity predicted changes in both BADLs and IADLs across 20 years (estimate=0.518, SE=0.153, p=.016; and estimate=0.656, SE=0.200, p<.001, respectively). Individuals who increased more in their stress reactivity across the 20 year period also showed greater increases in their functional health limitations.

